# Combined score of pretreatment platelet count and CA125 level (PLT-CA125) stratified prognosis in patients with FIGO stage IV epithelial ovarian cancer

**DOI:** 10.1186/s13048-019-0544-y

**Published:** 2019-07-31

**Authors:** Jie-Ping Chen, Qi-Dan Huang, Ting Wan, Hua Tu, Hai-Feng Gu, Jun-Ya Cao, Ji-Hong Liu

**Affiliations:** Department of Gynecologic Oncology, State Key Laboratory of Oncology in South China, Collaborative Innovation Center for Cancer Medicine, Sun Yat-sen University Cancer Center, Guangzhou, 510060 People’s Republic of China

**Keywords:** Epithelial ovarian cancer, Thrombocytosis, CA125, Inflammation, Immune evasion, Prognosis

## Abstract

**Background:**

The majority of death-related ovarian cancer is epithelial ovarian cancer (EOC). Regarding the Federation of Gynecology and Obstetrics (FIGO) stage IV EOC, the 5-year overall survival (OS) has not changed in last decades. Platelet (PLT) count and CA125 level are both prognostic markers that related to inflammation and immune evasion in EOC. This study intended to assess the prognostic value of pretreatment PLT count and CA125 level in FIGO stage IV EOC.

**Methods:**

The study included 108 patients diagnosed with FIGO stage IV EOC and treated with surgery and/or chemotherapy between January 1995 and December 2016. The PLT counts and CA125 levels of the patients before any treatment were analysed with clinical and pathological parameters, OS and progression-free survival (PFS). The survival of different groups was analyzed using the Kaplan-Meier method. The PLT-CA125 scores (0, 1, and 2) were defined basing on the presence of thrombocytosis (PLT count > 400,000/μL), an elevated CA125 level (CA125 > 1200 U/mL), or both. The prognostic value of PLT-CA125 was assessed with a Cox regression model.

**Results:**

Median OS, but not median PFS, was significantly decreased in patients with thrombocytosis or elevated CA125 levels when compared with the others (*p* = 0.011 & *p* = 0.004). The median OS was significantly decreased in patients with a PLT-CA125 score of 2 [37.8 months; 95% confidence interval (CI) 20.6–54.9] compared with patients with a PLT-CA125 score of 0 (70.0 moths, 95% CI 38.0–101.9, *p* < 0.001). The median PFS was also significantly decreased in patients with a PLT-CA125 score of 2 (19.6 months; 95% CI 13.0–26.3) compared with patients with a PLT-CA125 score of 0 (32.0 months; 95% CI 23.3–40.7, *p* = 0.011). Furthermore, multivariate analysis identified both PLT-CA125 scores of 2 and 1 as independent poor prognostic factors for OS (*p* = 0.004 & *p* < 0.001) and PFS (*p* = 0.033 & *p* = 0.017) compared with a PLT-CA125 score of 0.

**Conclusions:**

The pretreatment PLT-CA125 score can be a reliable marker with high accessibility for stratifying prognosis in patients with FIGO stage IV EOC.

## Introduction

Epithelial ovarian cancer (EOC), a lethal gynecologic cancer, accounts for 90% of ovarian malignancies [1, 2]. However, EOC has a high case-fatality ratio among gynecologic cancers [3]. EOC is larruping for distinct tumor biology of different histological types and the absence of anatomic barriers. [4] High grade serous carcinoma (HGSC) is the most common histological type with higher malignancy in EOC [3, 4]. The established treatment strategy for advanced EOC includes cytoreductive surgery and chemotherapy. Approximately 70% of EOC patients are diagnosed with advanced Federation of Gynecology and Obstetrics (FIGO) stage III or even higher stage [5]. Although overall survival (OS) has increased over the last decades in stage III EOC, survival of patients with FIGO stage IV has not changed [6]. The prognostic factors of EOC including FIGO stage, age, histological type, performance status, and location of metastases, that predict survival indicate different tumor biology and pave the way for individualization of therapy [7]. However, most prognostic factors were not studied specifically in stage IV EOC patients. There is an urgent need for stratifying prognosis in patients with stage IV EOC.

Thrombocytosis [platelet (PLT) count > 400,000/μL] is associated with various cancers. The rate of thrombocytosis ranges from 31 to 42% in EOC [8]. Thrombocytosis is identified as a prognostic factor in many retrospective studies of EOC [9]. The increase of platelet count is due to tumor-secreted cytokines, such as interleukin (IL-6), which plays a role in stimulating the growth of megakaryocytes and thrombocytosis [10]. IL-6 is overproduced in a variety of malignancies and is related to inflammation and immune suppression [11]. However, it is still unclear whether the poor survival of patients with thrombocytosis is caused by IL-6 itself or is a result of IL-6-induced thrombocytosis [12].

CA125 is an extensively studied tumor marker in EOC. The level of CA125 is used in screening test, diagnosis, monitoring of efficacy during chemotherapy, and management of follow up [13, 14]. The dynamic changes of CA125 levels at diagnosis and during chemotherapy were associated with chemosensitivity of drugs and new agents, tumor burden, and time of relapse [15, 16]. In addition, researchers found that glycogen CA125 of tumor cells binds to natural killer (NK) cells and is conducive to immune evasion [17]. As CA125 has been studied in EOC for decades, there are still many puzzles.

Stage IV EOC is a systemic disease presenting with parenchymal metastases and metastases of extra-abdominal organs [5]. The poor survival of patients with thrombocytosis was considered to be a result of IL-6-induced thrombocytosis [12]. Currently, the relevance of PLT count and CA125 level was found in EOC [18]. However, the prognostic values of both markers were not comparatively studied. Hence, we combined these markers into a PLT-CA125 score and assessed the prognostic value of this new marker in FIGO stage IV EOC.

## Materials and methods

### Patients

This study was approved by the institutional review board of Sun Yat-Sen University Cancer Center (2017-FXY-104). The study included patients who were clinically diagnosed with FIGO stage IV primary invasive ovarian-, fallopian tube-, or peritoneal cancer; who are treated with debulking surgery or chemotherapy between January 1995 and December 2016; and who had complete clinical data in the medical record system. Patients were excluded due to (1) histologically reported ovarian tumors other than EOC; (2) serious performance status that contraindicated surgery or platinum-based chemotherapy; and (3) known congenital thrombophilia, deep venous thrombosis, anticoagulant treatment, or pregnancy within 6 months at diagnosis.

**Fig. 1:**
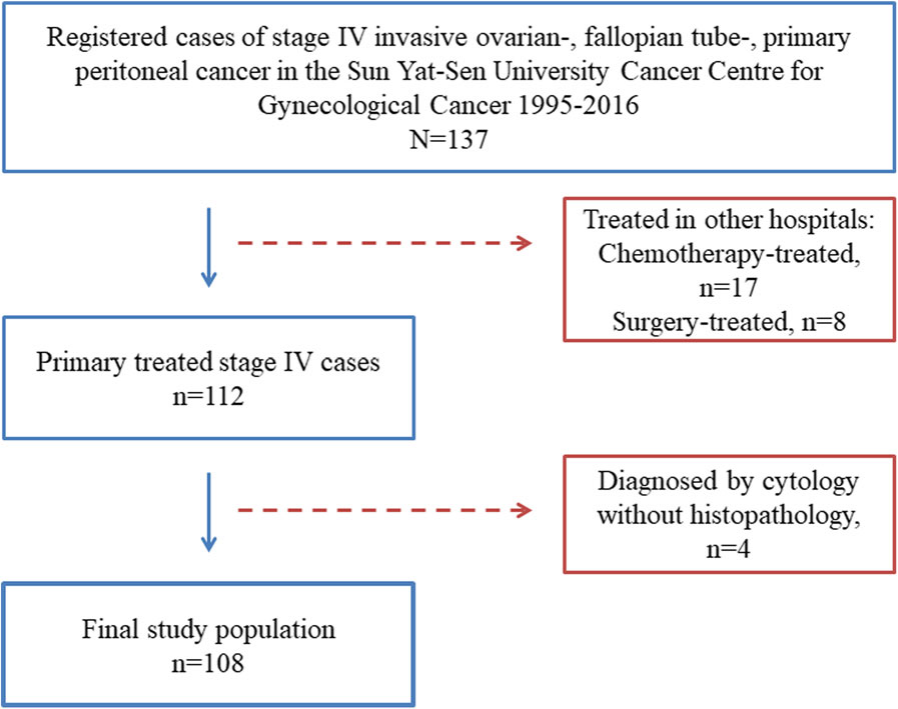
Inclusion flowchart of the study

Finally, 137 patients were included. Twenty-five Patients treated before operative pathological staging in other hospitals were excluded due to absence of pretreatment PLT count and CA125 level. Tumor staging was conformed based on the FIGO guidelines and four patients were excluded based on only cytology without definite histopathology [5]. As a result, a final study population comprised of 108 patients was used for prognostic analysis. (Fig. 1).

These patients were treated with primary debulking surgery (PDS) followed by adjuvant chemotherapy, neoadjuvant chemotherapy (NACT) followed by interval debulking surgery (IDS), or chemotherapy alone. Debulking surgery was classified as “optimal” if all visible lesions were resected during surgery. In addition to surgery, all patients had two and more cycles of platinum-based chemotherapy. Patients’ characteristics including age at diagnosis, histology, metastatic sites, results of complete blood cells counts and CA125 level were collected in medical record system. All patients were followed up annually with gynecological examination, pelvic and abdominal examinations, and tumor marker evaluation.

### Criteria of PLT and CA125

PLT count > 400 × 10^3^/μL was defined as thrombocytosis. The cut-off value for elevated CA125 levels (> 1200 U/mL) was determined from the receiver operating characteristic analysis. The PLT-CA125 score was defined as 0, 1, or 2 basing on the presence of thrombocytosis, elevated CA125 level, or both.

### Statistical analysis

All variables in the lattice table were analyzed using Pearson’s chi-square test, one-way ANOVA and Fisher’s exact test. Survival was analyzed using the Kaplan-Meier method. The end points of the study were OS and progression-free survival (PFS). The time length of PFS was from time of diagnosis to progressive disease or relapse. OS was defined as the length from time of diagnosis to death or the last follow-up. Survival data of patients alive without progression or those who died due to other disease were censored. In the Cox regression model, only variables that were statistically significant in univariate analysis were further analyzed in the multivariate analysis. In addition, Harrell’s C-index was calculated to evaluate the goodness fit of the Cox model. Software of statistical analyzes included STATA (ver. 20.0; Stata Corp, College Station, TX, USA), SPSS (ver. 13.0; SPSS Inc., Chicago, IL, USA), and R statistical software (R Foundation for Statistical Computing, Vienna, Austria). In this study, *p* < 0.05 was considered statistically significant.

## Results

### Patients characteristics

The study population included 108 patients with FIGO stage IV EOC treated in our hospital (Fig. 1). The median age of these patients was 51 years, ranging from 27 to 75 years. The most prevalent histological type was HGSC (85/108, 78.7%). Seventeen (17/108, 15.4%) patients had FIGO stage IVA disease, which is pleural effusion with positive cytology. In terms of treatment, 105 (97.22%) patients were treated with surgery. PDS was conducted in 53 patients (53/108, 49.1%). NACT and subsequent IDS treatment were performed in 52 patients (52/108, 48.1%). Three patients were treated with chemotherapy alone. The primary regimen of chemotherapy was paclitaxel-platinum combined chemotherapy.

Statistical descriptions of the patients’ characteristics is shown in Table 1. There is no significant difference detected between groups of patients with different PLA-CA125 scores, with the exception of metastatic pattern.

**Table 1:** Patients’ characteristics of PLT-CA125 scores in FIGO stage IV ovarian cancer

Variables	Total (*n* = 108)	PLT-CA125 score			*p*-value^a^
		0 (*n* = 39, %)	1 (*n* = 48, %)	2 (*n* = 21, %)	
Median age, years (range)	51 (27–75)	51 (33–75)	53 (27–72)	50 (38–69)	0.664
Age, n (%)					0.395
< 55	64 (59)	19 (49)	25 (52)	14 (54)	
≧55	44 (41)	20 (51)	23 (48)	7 (46)	
Histology, n (%)					0.709
HGSC	85 (79)	29 (74)	39 (81)	17 (81)	
Non-HGSC	23 (21)	10 (26)	9 (49)	4 (19)	
Primary treatment, n (%)					NA
PDS + chemotherapy	53 (49)	21 (54)	24 (50)	8 (38)	
NACT+IDS	52 (48)	17 (44)	22 (46)	13 (62)	
Chemotherapy alone	3 (3)	1 (3)	2 (4)	0 (0)	
Surgery, n (%)					0.760
Optimal surgery	82 (76)	30 (77)	35 (73)	17 (81)	
Suboptimal surgery/no surgery	26 (24)	9 (23)	13 (27)	4 (19)	
Metastatic pattern, n (%)					0.012
Pleural effusion with positive cytology	17 (16)	5 (13)	7 (15)	5 (24)	
LNM	44 (41)	10 (26)	21 (44)	13 (62)	
Other	47 (43)	24 (61)	20 (41)	3 (14)	
Follow up, n, median months (inerquartile range)					NA
Alive	49	23	20	6	
	39.7 (31.7–52.8)	45.7 (36.5–64.8)	37.1 (26.1–52.3)	33.5 (30.6–42.0)	
Dead	59	16	28	15	
	29.0 (19.7–45.3)	37.8 (9.9–64.8)	29.3 (20.0–43.1)	27.4 (19.7–41.7)	
Total	108	39	48	21	
	36.6 (23.4–48.1)	45.7 (29.9–64.8)	34.6 (21.9–43.8)	30.6 (24.4–41.2)	

Abbreviations: *PLT* platelet, *HGSC* high grade serous carcinoma, *Non-HGSC* non-high grade serous carcinoma, *NA* not applicable, *PDS* primary debulking surgery, *NACT* neoadjuvant chemotherapy, *IDS* interval debulking surgery, *LNM* lymph nodes metastasis beyond abdominopelvic cavity

^a^Tested by one-way ANOVA, Person chi square test or Fisher’s exact test, where applicable

### Survival analyzes of PLT count, CA125 level, and the PLT-CA125 score

**Fig. 2:**
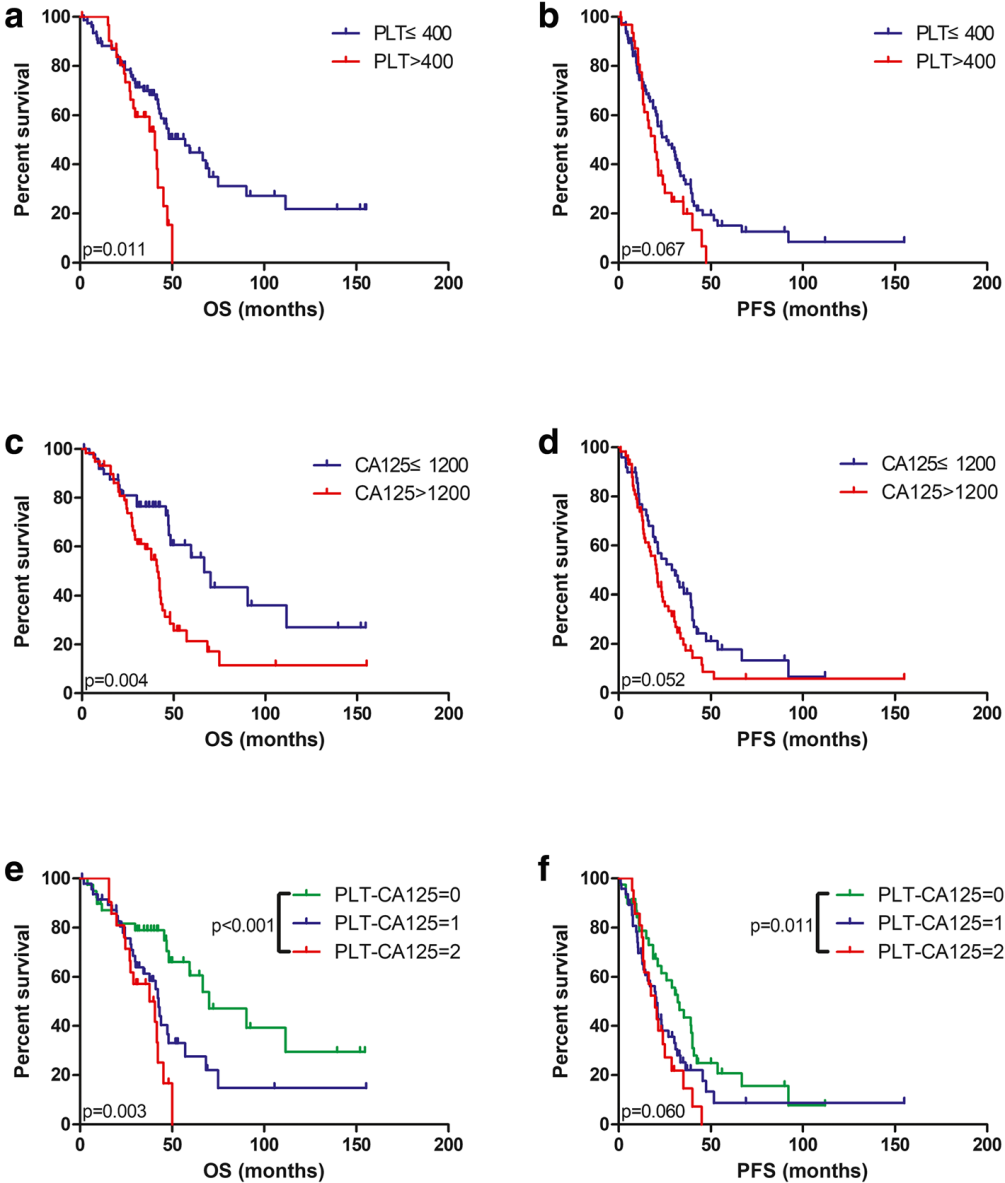
Survival curves of patients stratified by pretreatment PLT count and CA125 level. Kaplan-Meier curves for OS and PFS according to PLT count (**a** & **b**), CA125 level (**c** & **d**) and PLT-CA125 score (**e** & **f**)

Thrombocytosis (PLT > 400,000/μL) or an elevated CA125 level (CA125 > 1200 U/mL) was significantly associated with poorer OS in patients with stage IV EOC (Fig. 2a & c). The appropriate cut-off value of CA125 was calculated from the receiver operating characteristic curve analysis. However, both thrombocytosis and an elevated CA125 level were likely to be associated with shorter PFS without statistical significance (Fig. 2b & d). OS was shorter in patients with thrombocytosis than in those without thrombocytosis (40.0 months vs. 57.0 months, *p* = 0.011; Fig. 2a). PFS was decreased in patients with an elevated CA125 level when compared with patients who had a relatively low CA125 level (66.6 months vs. 41.0 months, *p* = 0.003; Fig. 2c).

As the associations of PLT count and CA125 level with OS were significant, we further calculated the association of the PLT-CA125 score with OS and PFS. A PLT-CA125 score of 2, indicating the presence of thrombocytosis and an elevated CA125 level, was associated with a worse median OS [37.8 months; 95% confidence interval (CI) 20.6–54.9] than that of a PLT-CA125 score of 0 (70.0 months, 95% CI 38.0–101.9, *p* < 0.001; Fig. 2e). Interestingly, PFS was also significantly decreased in patients with a PLT-CA125 score of 2 (19.6 months; 95% CI 13.0–26.3) compared with those with a PLT-CA125 score of 0 (32.0 months; 95% CI 23.3–40.7, *p* = 0.0115; Fig. 2f). These findings imply that the PLT-CA125 score can stratify PFS better than both markers alone.

### Cox proportional hazards model of FIGO stage IV EOC

The results of the univariate and multivariate Cox regression analyzes for OS and PFS are shown in Table 2. Univariate analysis of the Cox model for OS identified histological type other than HGSC, suboptimal surgery or no surgery, and a PLT-CA125 score of 1 or 2 as prognostic factors. Whereas NACT subsequent IDS or chemotherapy alone, suboptimal surgery or no surgery, and PLT-CA125 scores of 1 or 2 were associated with worse PFS. Multivariate Cox regression analysis identified PLT-CA125 scores of 1 [hazard ratio (HR) 2.590; 95% CI 1.356–4.946, *p* = 0.004] and 2 (HR 4.300; 95% CI 1.976–9.358, *p* < 0.001) as independent poor prognostic factors for OS. PLT-CA125 scores of 1 and 2 were also independent poor prognostic factors for PFS.

**Table 2:** Univariate and multivariate Cox models for OS and PFS

Variables	OS						PFS					
	Univariate			Multivariate			Univariate			Multivariate		
	HR	95% CI	*p*	HR	95% CI	*p*	HR	95% CI	*p*	HR	95% CI	*p*
Age (yr)												
< 55	Reference						Reference					
≧55	1.154	0.686–1.939	0.589				0.933	0.608–1.431	0.751			
Histology, n (%)												
HGSC	Reference			Reference			Reference					
Non-HGSC	1.840	1.053–3.216	0.032	2.121	1.196–3.764	0.010	1.356	0.813–2.264	0.244			
Primary treatment, n (%)												
PDS + chemotherapy	Reference						Reference			Reference		
NACT+IDS/ Chemotherapy alone	1.386	0.819–2.346	0.224				1.659	1.068–2.577	0.024	1.571	1.010–2.444	0.045
Surgery, n (%)												
Optimal surgery	Reference			Reference			Reference					
Suboptimal surgery/no surgery	2.167	1.275–3.681	0.004	2.246	1.314–3.840	0.003	1.763	1.077–2.886	0.024	2.086	1.249–3.486	0.005
Metastatic pattern, n (%)												
Pleural effusion with positive cytology	Reference						Reference					
LNM	0.864	0.421–1.772	0.689				1.291	0.673–2.477	0.443			
Others	0.527	0.251–1.105	0.090				0.822	0.424–1.597	0.564			
PLT-CA125												
0	Reference			Reference			Reference			Reference		
1	2.203	1.178–4.119	0.013	2.590	1.356–4.946	0.004	1.553	0949–2.540	0.080	1.751	1.047–2.929	0.033
2	3.580	1.692–7.576	0.001	4.300	1.976–9.358	< 0.001	2.015	1.108–3.665	0.022	2.131	1.146–3.960	0.017

Abbreviations: *OS* overall survival, *PFS* progress-free survival, *HR* hazard ratio, *CI* confidence interval, *HGSC* high grade serous carcinoma, *Non-HGSC* non-high grade serous carcinoma, *PDS* primary debulking surgery, *NACT* neoadjuvant chemotherapy, *IDS* interval debulking surgery, *LNM* lymph nodes metastasis.beyond abdominopelvic cavity, *PLT* platelet

Furthermore, Harrell’s C-index of the Cox proportional hazards model that included the PLT-CA125 score (C-index: 0.684 for OS; 0.622 for PFS) was relatively higher than that of the model without the PLT-CA125 score (C-index: 0.633 for OS; 0.581 for PFS).

### Subgroup analysis of PLT-CA125 score

To identify the specific clinical factors related to survival, we also assessed the prognostic value of the PLT-CA125 score grouped by FIGO stage, age, histology, primary treatment, and surgical satisfaction (Table 3). There was a significant association between PLT-CA125 and OS with regard to the factors of stage (*p* = 0.003 for stage IVB), age (*p* = 0.003 for < 55), histology (*p* = 0.002 for HGSC), treatment (*p* = 0.009 for PDS), and surgical satisfaction (*p* = 0.003 for optimal surgery).

**Table 3:** Subgroup analysis of prognostic factors

Groups	PLT-CA125	N (%)	OS (months)			PFS (months)		
			Median (SD)	95% CI	*p*	Median (SD)	95% CI	*p*
FIGO stage								
IVA	0	5 (29.4)	48.3 (14.6)	19.6–77.0	0.600	11.17		0.185
	1	7 (41.2)	47.4 (13.4)	21.1–73.7		31.8 (13.6)	5.14–58.5	
	2	5 (29.4)	41.7 (10.1)	21.9–61.5		13.3 (5.59)	2.32–24.2	
IVB	0	34 (37.4)	90.4 (18.9)	53.2–127.6	0.003	32.0 (4.37)	23.4–40.6	0.107
	1	41 (45.1)	42.7 (2.99)	36.8–48.5		20.0 (3.12)	13.9–26.1	
	2	16 (17.6)	37.8	21.2–54.4		19.7 (3.15)	13.5–25.8	
Age								
< 55	0	19 (32.8)	90.4 (29.2)	33.2–147.6	0.003	35.4 (8.95)	17.9–52.9	0.101
	1	25 (43.1)	42.7 (0.71)	41.3–44.1		21.0 (2.89)	15.4–26.6	
	2	14 (24.1)	40.7 (8.2)	24.7–47.1		16.2 (3.98)	8.43–24.0	
≧55	0	20 (40.0)	70.0 (13.0)	44.6–95.4	0.183	32.0 (6.29)	19.7–44.3	0.404
	1	23 (46.0)	28.7 (9.13)	10.8–46.6		16.9 (5.01)	7.11–26.7	
	2	7 (14.0)	37.8 (8.34)	21.5–54.1		23.9 (3.45)	18.2–28.4	
Histology								
HGSC	0	29 (34.1)	111.6 (26.6)	59.4–163.8	0.002	35.4 (4.45)	26.69–44.1	0.021
	1	39 (45.9)	42.7 (4.88)	33.1–52.2		20.0 (4.61)	10.9–29.0	
	2	17 (20.0)	41.7 (2.77)	36.3–47.1		21.5 (2.82)	18.9–27.0	
Non-HGSC	0	10 (43.5)	20.4 (20.5)	0.00–60.5	0.189	9.57 (11.4)	0.00–31.9	0.117
	1	9 (38.3)	43.2 (5.55)	32.3–54.1		33.5 (15.8)	2.52–64.5	
	2	4 (17.4)	15.6			8.83 (2.08)	4.75–12.9	
Primary treatment								
PDS + chemotherapy	0	21 (39.6)	90.4 (31.5)	28.7–152	0.009	40.1 (3.71)	32.8–47.3	0.104
	1	24 (45.3)	42.7 (6.04)	30.8–54.5		21.1 (4.42)	12.4–29.8	
	2	8 (15.1)	40.7 (15.7)	9.87–71.5		17.5 (5.47)	6.79–28.25	
NACT+IDS/ Chemotherapy alone	0	18 (32.7)	70.0 (29.1)	12.9–127	0.254	23.3 (4.97)	13.6–33.0	0.571
	1	24 (45.3)	43.2 (3.87)	35.6–50.8		20.0 (2.68)	14.74–25.26	
	2	13 (23.6)	37.8 (7.67)	22.8–52.8		19.7 (4.46)	10.9–28.4	
Surgery								
Optimal surgery	0	30 (36.6)	80.4 (20.2)	50.8–130	0.003	32.0 (6.14)	19.9–44.0	0.167
	1	35 (42.7)	43.9 (3.21)	37.6–50.2		23.6 (6.23)	11.4–35.8	
	2	17 (20.7)	40.7 (10.2)	20.8–60.6		21.5 (6.07)	11.6–31.4	
Suboptimal surgery/no surgery	0	9 (34.6)	45.7 (23.6)	0.00–91.86	0.175	35.4 (17.4)	1.37–69.5	0.046
	1	13 (50.0)	23.2 (13.5)	0.00–49.7		12.8 (4.26)	4.49–21.2	
	2	4 (15.4)	37.8 (12.4)	13.6–62.0		12.6 (4.68)	3.44–21.8	

Abbreviations: *PLT* platelet, *OS* overall survival, *PFS* Progress-free survival, *SD* standard deviation, *CI* confidence interval, *HGSC* high grade serous carcinoma, *Non-HGSC* non-high grade serous carcinoma, *PDS* primary debulking surgery, *NACT* neoadjuvant chemotherapy, *IDS* interval debulking surgery, *LNM* lymph nodes metastasis beyond abdominopelvic cavity

The PFS of patients with different PLT-CA125 scores, which were 0, 1, and 2, were significantly different in the subgroups of patients with histology of HGSC (*p* = 0.021) and those who received suboptimal surgery or no surgery (*p* = 0.046).

## Discussion

In our study, the PLT-CA125 score was an independent prognostic factor for both OS and PFS in stage IV EOC. However, neither thrombocytosis nor elevated CA125 level was significantly associated with short PFS. The survival for stage IV patients can be more specifically stratified using combined PLT-CA125 scoring model compared with using PLT or CA125 alone. These findings imply that both thrombocytosis and an elevated CA125 level may equally contribute to the poor survival of patients with stage IV EOC.

Currently, there are evidences from retrospective studies that suggest a potential relationship between platelet count and CA125 level [19]. Platelet is an important modulator in many physiological functions of cancer, and thrombocytosis is identified as a prognostic factor in many studies of EOC [20]. IL-6, which is commonly elevated in patients with EOC and is known to be an important cytokine of inflammation and immune suppression, can stimulate megakaryocyte growth and thrombopoiesis [8]. Platelets take part in the process of haematogenous metastasis by parcelling tumor cell in the vasculature system [21]. On the other hand, platelets can promote tumor cell metastasis by breaking the membrane of vessels through the release of enzymes [22]. In addition, tumor cells expressing glycogen can aggregate platelets and induce tumor-platelet aggregation, which help tumor cells survive from immune clearance [21]. Recently, the dual role of platelets in immune functions and inflammation has gained more and more attention in infectious disease and cancer [23].

Inflammation plays an important role of tumorgenesis and tumor progression in EOC, especially in advanced disease [24, 25]. At the same time, inflammatory cytokine, IL-6, can stimulate megakaryocyte growth and induce thrombocytosis [8]. Although EOC is considered unresponsive to immune therapy, there are increasing evidences suggesting that EOC is, in fact, an immunogenic tumor with highly heterogeneous subtypes [26]. Diverse clinical and epidemiological data have shown that a natural antitumor immune response of tumor infiltrating lymphocytes and NK cells in EOC [27]. Emerging evidence about inflammation and tumor immune suppression mechanisms may pave the way for immune therapy.

Over the last decades, CA125 is extensively investigated and widely used in the diagnosis and follow-up of EOC [18]. In addition, elevated CA125 is noted in inflammatory diseases and several benign diseases. Expression of CA125 in EOC plays important roles in cell growth, transformation, and invasion of tumor cells [28]. As a large glycoprotein, the CA125 glycogen is an important molecule contacting with other cells, including NK cells and fibroblasts [17]. The immune evasion mechanism of NK cell suppression through binding to CA125 has been reported in EOC [29]. Both CA125 and platelets are related to inflammation and play important roles in the pathological status of immune surveillance in EOC. In our study, we combined these markers as a novel scoring model to stratify OS and PFS in stage IV EOC, which were shown to be better than using PLT or CA125 alone. This result implies that the PLT-CA125 score, reflecting inflammation and immune suppression, could be used to predict survival and determine potential therapeutic strategies targeting inflammatory and immune surveillance in advanced EOC.

In addition to the prognostic significance of the PLT-CA125 score, the high accessibility makes it practical in clinical use. Platelets count and CA125 level can be easily obtained in all patients suspected with ovarian cancer.

Stage IV EOC is a systemic disease and is often diagnosed with parenchymal metastases and metastases of extra-abdominal organs [5]. Our study demonstrated that the combined score of systemic inflammatory marker, PLT, and EOC tumor marker CA125 was a reliable prognostic factor for stage IV EOC. In addition, other prognostic factors of stage IV EOC, including histological type and satisfaction of surgery, were demonstrated in our study.

As a retrospective study, there are several limitations. Firstly, the sample size of the study was relatively small. Thus, confirmation of results in other cohorts and centers is necessary for further research. Secondly, the cut-off value for CA125 may vary in different study cohorts. Although the cut-off value of CA125 has been confirmed in our study cohort, the value may vary in different cohorts and centers. Finally, the performance statuses of the patients were not included in our study, which may affect the daily life abilities of the patients, and help determine treatment strategies, as well as prognosis. However, this is an inevitable defect of retrospective study.

In conclusion, the PLT-CA125 score is an independent prognostic factor in patients with stage IV EOC. It is a useful and highly accessible marker for predicting clinical outcomes and suggesting for potential therapeutic strategies in patients with stage IV EOC.

## Data Availability

The datasets generated in this study are available in the Research Data Deposit. (http://www.researchdata.org.cn), with the approval number of RDDA2019001031.

## Abbreviations

CI: Confidence interval
HGSC: High grade serous carcinoma
HR: Hazard ratio
IDS: Interval debulking surgery
LNM: Lymph node metastasis beyond the abdominopelvic cavity
NA: Not applicable
NACT: Neoadjuvant chemotherapy
Non-HGSC: Non-high grade serous carcinoma
OS: Overall survival
PDS: Primary debulking surgery
PFS: Progression-free survival
PLT: Platelet
SD: Standard deviation
